# Optimising neonatal bubble continuous positive airway pressure: A Somaliland quality initiative

**DOI:** 10.4102/phcfm.v17i1.4742

**Published:** 2025-04-23

**Authors:** Hawa D. Mahmoud, Sarah C. Kent, Fatima E. Ibrahim, Najma Mohamed, Fatima A. Abdulahi, Meagan N. O’Neal, Priya Kanajam, Ellen K. Diego

**Affiliations:** 1 Department of Family Medicine, College of Health Sciences, Amoud University, Borama, Somalia; 2 Department of Neonatal-Perinatal Medicine, University of Minnesota, Minneapolis, United States; 3 College of Medicine, University of Minnesota, Minneapolis, United States; 4 Division of Neonatal-Perinatal Medicine, Department of Pediatrics, University of Minnesota, Minneapolis, United States

**Keywords:** quality improvement, neonatal respiratory distress, bubble continuous positive airway pressure, low- and middle-income countries, neonatal mortality, neonatal intensive care unit

## Abstract

**Background:**

Continuous positive airway pressure (CPAP) therapy is the standard of care for neonatal respiratory distress and improves survival when implemented in low-resource settings. Clinical audits at the Borama Regional Hospital (BRH) Neonatal Intensive Care Unit (NICU) revealed multiple barriers to effective CPAP, including insufficient pressure, a lack of neonatal-sized nasal prongs, and patient interface challenges.

**Aim:**

Improve respiratory distress by increasing effective CPAP delivery for neonates < 30 days of age from 52% to 90% in 6 months.

**Setting:**

Single-centre referral hospital in the Awdal region of Somaliland.

**Methods:**

Quality improvement (QI) initiative with outcomes displayed using statistical process control (SPC) charts.

**Results:**

Eleven residents, three medical interns and seven NICU nurses completed the educational training. Forty-five patients were initiated on the locally designed bubble CPAP (bCPAP) device with a 47% (122/261) CPAP safety checklist completion rate for the three daily nursing shifts. We achieved our study aim by increasing the adherence rate to the 7-item bCPAP device set up from a baseline of 52% to 91%. The rate of infants weaned or discontinued from bCPAP for improved respiratory severity score (RSS) increased from 0% to 18% but did not demonstrate process change. There was no increase in adverse event rates (air leak, nasal columella breakdown and nasal irritation).

**Conclusion:**

We demonstrated increased effective bCPAP delivery and decreased respiratory distress.

**Contribution:**

This study outlines low-cost, customisable QI strategies to address commonly encountered gaps for effective bCPAP delivery in low-resource settings without access to commercially available CPAP devices or speciality-trained providers.

## Introduction

Ninety-nine percent of global neonatal mortality occurs within low- and middle-income countries (LMICs) and sub-Saharan Africa remains the region with the highest neonatal mortality rate in the world.^1,2,3^ The Republic of Somaliland is a self-declared sovereign state in the Horn of Africa that is considered internationally as part of Somalia. In Somaliland, 33% of deaths under-five occur in the neonatal period, which remains well above the 2030 Sustainable Development Goal (SDG) of < 12 neonatal deaths per 1000 live births.^4^ A 2022 multi-centre retrospective analysis of Neonatal Intensive Care Unit (NICU) patients admitted at Somali Regional State public hospitals in Eastern Ethiopia reported prematurity (44.6%), low birth weight (33.5%), and birth asphyxia (27.6%) as the leading causes of death.^5,6,7^

Neonatal respiratory distress contributes significantly to early neonatal morbidity and mortality in both term (e.g. pneumonia, transient tachypnea of the newborn [TTN], meconium aspiration syndrome [MAS], pneumothorax) and preterm infants (e.g. respiratory distress syndrome [RDS], apnoea of prematurity, bronchopulmonary dysplasia).^8,9,10,11^ A common and dangerous side effect of preterm birth is RDS, which is brought on by a surfactant shortage and structural immaturity of the lungs.^12^ Respiratory distress syndrome treatment methods have significantly increased survival rates in high-income nations and include continuous positive airway pressure (CPAP), mechanical ventilation, and exogenous surfactant replacement therapy.^12^ In resource-limited settings, lack of availability may prevent the implementation of some of these more advanced clinical interventions that have been shown to reduce respiratory morbidity and mortality.^9^

For the treatment of newborns experiencing respiratory distress, CPAP therapy has been shown to reduce mortality when implemented in low-resource settings.^13,14,15,16,17,18^ Numerous studies have demonstrated that early CPAP is as effective as surfactant therapy or invasive mechanical ventilation and can effectively treat RDS in preterm infants.^19^ Leading international health organisations, such as the World Health Organization (WHO), recommend CPAP as the standard of care for use in low-resource settings.^13,20^

Bubble CPAP (bCPAP) is a low-cost, non-invasive form of CPAP that requires less technical expertise to operate, avoids ventilator-induced lung injury, stimulates lung growth, and has a lower risk of patient complications when compared with mechanical ventilation.^16,21,22,23^ Therapy with bCPAP lessens the requirement for mechanical ventilation by applying a constant distending pressure to maintain alveolar expansion, reduce work of breathing and improve functional residual capacity.^19,24^ There are a variety of commercial bCPAP devices specifically designed to be easily maintained and reasonably priced.^18,22,25^ For these reasons, it is an appealing treatment option for use in low-resource settings.^26^ Despite the many advantages, there are many barriers to delivering effective bCPAP, including the need for electricity, compressed air, oxygen blending systems, and the availability of skilled staff for training, maintenance and patient monitoring.^15,20^

Amoud University (AU) is Somaliland’s first institution of higher education founded in Borama.^27,28^ In the Borama District, there are 13 health centres and one 377-bed regional hospital (BRH) staffed by AU Family Medicine (FM) physicians.^27^ Unpublished data from a retrospective chart review conducted by AU FM physicians in 2015 revealed RDS (15%), pneumonia (38%) and apnoea of prematurity (26%) as key causes of respiratory morbidity for neonates born at BRH. The BRH NICU was subsequently established by AU FM physicians in 2017 to improve neonatal mortality and respiratory care provision. In 2020, the Somaliland government implemented a policy to provide free obstetric care for all patients referred to delivery at BRH from the district health centres.^27^ This policy change led to a rapid rise in the number of deliveries occurring at BRH and an increase in the number of annual NICU admissions (264 NICU admissions and an 85% survival rate).^27^

Facility-level gap assessments using quality improvement (QI) methodology have been an effective strategy to improve adherence to the standard of care for respiratory management in neonates.^18,29,30,31^ To better qualify the current gaps in best practise standards, clinical audits were implemented and supervised by a US-trained neonatal-perinatal medicine physician at the BRH NICU in August 2022 prior to the implementation of this QI initiative. These clinical audits identified multiple gaps in the efficacy of the locally designed bCPAP device setup and in the clinical evaluation of infants with respiratory distress. Gaps were observed in the following domains: respiratory assessment gaps (absence of clinical criteria or validated scoring system to determine need for CPAP), pressure delivery gaps (lack of ability to pressure-test device, lack of expiratory limb depth demarcation, and lack of water bottle seal to prevent evaporative water loss), patient interface gaps (lack of neonatal-sized nasal prongs, lack of cannula securement method, and lack of standardised patient assessment by bedside staff to ensure prongs remain in position), oxygen supply gaps (inability to blend oxygen due to lack of compressed air source), humidification gaps (humidification systems not attached in the respiratory circuit) and disinfection system gaps (routine reuse of single-use equipment without a system for disinfection).

In addition to the equipment and skill barriers, there is a significant geographic barrier to obtaining essential medical equipment. Somaliland’s independence has not been officially recognised internationally, leading to blocks on shipping and trade with surrounding countries. Currently, there is no formal mail delivery system in Somaliland. For that reason, there are significant limitations on the direct ordering and delivery of medical supplies from reputable manufacturers. Some equipment can be obtained indirectly through trade and imports from the nearest port city approximately 300 km from the clinical site. However, this can be quite costly, so locally sourced equipment is preferred. We aimed to overcome this barrier to care by providing a sustainable source of medical equipment that can be internationally supplied and locally maintained.

The global aim of this QI initiative was to expand staff knowledge and skills to improve bCPAP delivery and reduce preventable neonatal deaths in the Borama community. Educational interventions targeted knowledge gaps in bCPAP principles and the accuracy of respiratory distress identification. Skill interventions addressed strategies to monitor and troubleshoot common equipment and patient interface challenges.

The QI SMART (Specific, Measurable, Achievable, Relevant and Time-Bound) aim was to improve respiratory distress by increasing effective bCPAP for all neonates < 30 days of age admitted at BRH from 52% to 90% between August 2023 and December 2023.

## Research methods and design

### Study design

This project was designed as a single-centre quasi-experimental interrupted time series QI initiative using three Plan-Do-Study-Act (PDSA) cycles. This interrupted time series design required data collection at multiple time points before, during and after the introduction of study change ideas, which is outlined in the intervention portion of the methods section below.

### Setting

Borama Regional Hospital is the only referral hospital for high-risk deliveries in the Awdal region with a combined population of over 200 000. The NICU is staffed daily by two FM resident physicians, who are supervised by a FM or paediatric attending physician. An average of 22 patients per month are admitted to the unit. There are five nurses who staff the unit and provide continuous nursing coverage in two 7 h shifts and one 10 h shift (Shift A: 07:00–14:00; Shift B: 14:00–21:00 and Shift C: 21:00–7:00). Shift A has the most robust staffing model with an average of two nurses working daily, while shift B and C maintain one nurse per shift.

### Study population and sampling strategy

The study population was drawn from a convenience sample of any newborn infant < 30 days of age delivered by any type of delivery route (vaginal or caesarian section) at BRH. Inclusion criteria for participation included those infants demonstrating clinical signs and symptoms of respiratory distress in the first 30 days of life as evidenced by a Silverman Andersen respiratory severity score (RSS) of ≥ 5. Inclusion criteria also require the neonate to be admitted to the BRH NICU for initiation on bCPAP. We excluded any infant not surviving resuscitation efforts in the delivery room before admission to the NICU and newborns of parents or legal guardians who refused admission to the NICU. The participant sample size was not predetermined. Patient enrollment continued until the study endpoint, as defined by the QI SMART aim of increasing effective bCPAP from 52% to 90% between August 2023 and December 2023.

### Intervention

#### Period 1. Baseline data collection (June 2023–August 2023)

Baseline data collection occurred prospectively by auditing the current bCPAP system in the 2-month period (Period 1) preceding all process changes. Baseline data were collected using a REDCap (Research Electronic Data Capture) clinical audit survey.^32^ The survey included patient demographics (postnatal age, weight and sex), indication for bCPAP and an equipment checklist (equipment presence, functionality and appropriately disinfected). The audit also tracked the patient clinical status (signs and symptoms of respiratory distress), bCPAP device setup, effective pressure delivery, appropriate nasal prong placement, rate of air leak and nasal septal damage.

#### Period 2. Education and protocol development (August 2023)

**FIGURE 1:**
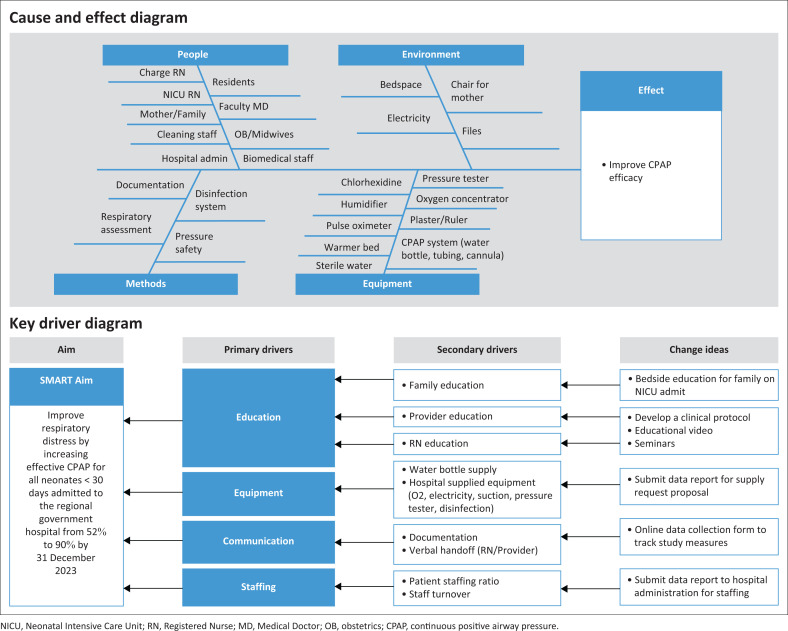
Cause and effect diagram and key driver diagram developed by the multidisciplinary quality improvement team to identify key drivers of change: (1) education; (2) equipment; (3) communication; (4) staffing.

**Quality improvement educational interventions:** In the pre-implementation phase, a multidisciplinary team composed of FM physicians, nurses and hospital administrators was assembled. The QI team received a 2-week training course in QI methodology from international collaborators through a combination of lecture-based instruction and skills training. The QI team was introduced to the QI toolkit available through the Institute for Healthcare Improvement (IHI)^33^, which includes rapid cycle feedback systems using the PDSA approach. The team generated a cause and effect diagram and key driver diagram to identify the following key drivers of change (Figure 1): (1) education; (2) equipment; (3) communication; and (4) staffing.

Key Driver 1: Education: *Change Idea 1: Develop a clinical protocol and educate bedside staff on how to implement the protocol*.

**FIGURE 2:**
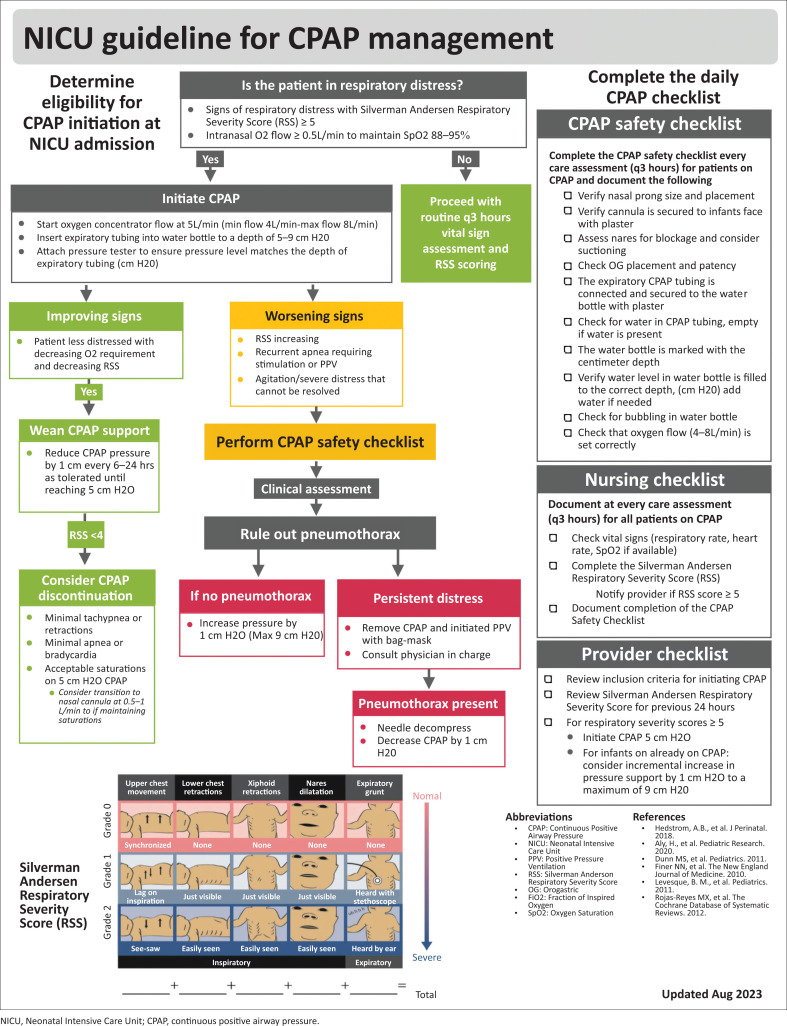
Borama Regional Hospital neonatal intensive care unit clinical guideline for continuous positive airway pressure management.

Clinical Protocol Development: During Period 2, the QI team and US-trained neonatal-perinatal medicine specialists conducted an evidence-based literature review to create a local clinical bCPAP protocol based on international guidelines. The BRH NICU bCPAP protocol incorporated educational, process and systems-based changes to target accurate identification of respiratory distress, equipment monitoring and device utilisation (Figure 2).

Bubble CPAP Educational Intervention: Provider and nursing education was conducted through a combination of lecture-based instruction and in-person skills training. Lecture-based training topics are outlined in Table 1. Staff completed skills training in the utilisation of the Silverman Andersen RSS to diagnose respiratory distress on admission and during clinical care. Additional skills workshops reviewed equipment setup, management and interface challenges. Attendance of teaching sessions was tracked. Post-assessment knowledge checks and observed structured clinical examinations were employed to assess knowledge accrual. Educating the family was also a part of the study purpose in which providers and nurses participated in counselling the families about the bCPAP and its importance. New NICU nurses hired during the implementation period were trained by the QI team.

**TABLE 1:** Post-training assessment score reports for the six education topics are displayed for Neonatal Intensive Care Unit staff participants.

Education Intervention	Average Score	Min Score	Max Score	s.d.	s.d. Pass Rate
Non-Invasive Ventilation (#/14)	74%	64%	79%	±6	100%
Neonatal Respiratory Distress (#/21)	85%	67%	95%	±10	100%
CPAP Set Up (#/4)	80%	50%	100%	±20	80%
Introduction to Quality Improvement Methodology (#/16)	78%	56%	94%	±13	92%
Introduction to Infection Control (#/16)	74%	56%	94%	±11	92%
Troubleshooting Common Problems with Non-Invasive Ventilation (#/14)	74%	64%	79%	±6	100%

**Patient Demographic Data**	**Baseline (*N* = 10)**		**Intervention (*N* = 45)**		***p* Value**

Sex (female), *n* (%)	5 (50%)		21 (47%)		0.85
Day of life at initation of CPAP, median day (IQR)	1 (1–1.3)		1 (1–1)		0.17
Current weight, mean (SD)	2.3 (1.3)		2.3 (0.9)		0.44
**Birth gestational age, *n* (%)**					
27-29 weeks	-		4 (9%)		-
30-32 weeks	-		10 (24%)		-
33-35 weeks	-		4 (9%)		-
36-38 weeks	-		13 (30%)		-
39-41 weeks	-		11 (26%)		-
≥ 42 weeks	-		1 (2%)		-

**Indication for CPAP**	**Baseline (*N* = 10)**		**Intervention (*N* = 60)**		***p* Value**

RDS, *n* (%)	8 (80%)		37 (62%)		0.24
TTN, *n* (%)	0 (0%)		10 (17%)		0.07
MAS, *n* (%)	4 (40%)		21 (35%)		0.76
Pneumonia, *n* (%)	0 (0%)		9 (15%)		0.08
Sepsis, *n* (%)	8 (80%)		48 (80%)		0.80
Air leak, *n* (%)	0 (0%)		0 (0%)		-
Apnoea, *n* (%)	3 (30%)		11 (18%)		0.41
HIE, *n* (%)	3 (30%)		12 (20%)		0.49
Cardiac disease, *n* (%)	1 (10%)		3 (5%)		0.56

**Study Intervention**	**Weaning on CPAP (*N* = 16)**		**Stable or Worsening on CPAP (*N* = 44)**		***p* Value**

RDS, *n* (%)	6 (38%)		31 (70%)		0.01
TTN, *n* (%)	5 (31%)		5 (11%)		0.04
MAS, *n* (%)	7 (44%)		14 (32%)		0.45
Pneumonia, *n* (%)	3 (19%)		6 (14%)		0.44
Sepsis, *n* (%)	13 (81%)		35 (80%)		0.85
Air leak, *n* (%)	0 (0%)		0 (0%)		-
Apnoea, *n* (%)	3 (19%)		8 (18%)		0.87
HIE, *n* (%)	2 (13%)		10 (23%)		0.30
Cardiac disease, *n* (%)	0 (0%)		3 (7%)		0.14

**Process Measure**	**Baseline**		**Intervention**		***p* Value**

CPAP audit form completion, *n* (%)	N/a		122/261 (47%)		-
CPAP set up (# correct/7-items), *n* (%)	43/70 (61%)		571/616 (93%)		0.01

**Outcome Measure**	**Baseline**		**Intervention**		***p* Value**

Weaning or discontinuing CPAP for improved RSS score, *n* (%)	0/10 (0%)		16/87 (18%)		0.14

**Balancing Measure**	**Baseline**		**Intervention**		***p* Value**

Adverse events (nasal irritation, columella breakdown, pneumothorax), *n* (%)	1/10 (10%)		6/87 (7%)		0.38

s.d., standard deviation; IQR, interquartile range; HIE, Hypoxic Ischemic Encephalopathy; N/a, not applicable; RDS, Respiratory Distress Syndrome; TTN, Transient Tachpnea of the Newborn; MAS, Meconium Aspiration Syndrome; CPAP, continuous positive airway pressure; RSS, Respiratory Severity Score.

Key Driver 2: Equipment: *Change Idea 2: Standardise bCPAP equipment setup.*

Bubble CPAP Equipment: Selected elements of the current locally designed CPAP device setup were exchanged to generate a novel locally designed bCPAP device (Online Appendix Figure 1-A1). The novel, modified and retained elements of the bCPAP setup with unit price are outlined below.

Novel bCPAP system elements:

- Patient interface: Adult-sized nasal prongs were replaced with neonatal-sized (preterm, late-preterm and term) RAM cannula ($14.50 per unit).
- Pressure delivery: Adult-size oxygen tubing was replaced with a large-diameter corrugated tubing breathing circuit ($28.70 per unit), which also required the use of oxygen tubing ($0.49 per unit) and step-down adapter ($0.84 per unit) for application.
- Pressure testing device.
- Nasogastric tube ($0.50 per unit).
- bCPAP Delivery: A labelling system for measuring expiratory limb submersion in a water bottle was developed using NICU-provided plaster (tape) and markers.

Modified bCPAP system elements:

- Humidification: Humidifier bottles not actively in use in the unit were disinfected and attached to the circuit.

Retained bCPAP elements:

- Oxygen concentrator: Two oxygen-concentrating devices currently in use in the NICU were maintained.
- Water bottles (350 mL: $0.25 per unit) continued to be purchased at the local market and provided by family members.

Total Cost: $45.28 USD per bCPAP device.

Disinfection system: A disinfection system was developed with hospital-supplied disinfection materials obtained from the local storage facility for reprocessing of reusable CPAP equipment (Online Appendix Figure 2-A1) and are outlined below:

- Step 1: Liquid soap water, plastic wash bucket, scrub brushes and gloves.
- Step 2: 0.5% Chlorhexidine solution, water, plastic wash bucket and gloves.
- Step 3: Clean water rinse.

Key Driver 3. Communication: *Change Idea 3. Improve communication and documentation.*

Quality improvement team members conducted training on documentation and verbal handoffs. A bCPAP safety checklist (Figure 2) was developed for completion by nursing staff during each shift (three checklists per day). Study measures (see Period 3 for a detailed list of study measures) were collected for the preceding 24 h period daily by NICU physician staff during bedside rounds using an online REDCap clinical audit survey for each patient on bCPAP therapy.

Key Driver 4. Staffing: *Change Idea 4. Improve patient-to-staff ratio and reduce staff turnover.*

To maximise care for patients, improving the patient-to-staff ratio and minimising staff turnover was discussed with BRH leadership and administration. A plan was developed to prepare a data report following the completion of the QI initiative to detail the unit-specific staffing needs.

#### Period 3. Study intervention (August 2023–December 2023)

In August 2023, the BRH NICU bCPAP Protocol was released for use by all NICU staff. Data collection occurred through the REDCap clinical audit tool described earlier, and included the following study measures.

### Patient demographics

Birth gestational age, postnatal age (days), weight, sex, and indication for initiation of bCPAP.

### Study measures

Educational measures included lecture-based education post-assessment scores and skills workshop completion rate. Process measures included bCPAP safety checklist completion rate and timing (minutes or hours of life) of bCPAP initiation. The process measures captured by the bCPAP safety checklist include the Silverman Andersen RSS, availability of bCPAP equipment (equipment present, functional and disinfected) and 7-item bCPAP setup checklist (correct nasal cannula size and position, nasal cannula secured in place, expiratory tubing connected and secured, water bottle depth marked, expiratory tubing submerged to desire bCPAP pressure, bubbling observed in water bottle, correct oxygen flow 5 L per minute – 8 L per minute set). Outcome measures included the rate of infants weaning or discontinuing bCPAP for improved RSS and death. Balancing measures included the rate of air leak (pneumothorax), nasal columella breakdown, and nasal irritation.

### Data analysis

Time series analysis was utilised for data analysis. Statistical process control (SPC) charts were employed to track study measures, determine progress towards achieving the study SMART aim, and identify special cause variation. Statistical process control charts were generated using QI Macros for Excel version 2020. Western Electric Rules were used to identify special cause variation. Centreline shifts and control limits were adjusted when meeting the criteria for special cause variation.

Demographic variables and selected study indicators were compared between the baseline study cohort (Period 1) and the intervention study cohort (Period 3). All variables were assessed for normalcy. A chi-squared (χ^2^) test was performed for nominal, unmatched data, and an independent t-test was performed for continuous, unmatched data. All continuous outcomes required Levene’s test for equality of variances. If significant variance occurred between groups, degrees of freedom were adjusted using the Welch-Satterthwaite method. Data analyses were performed using IBM SPSS Statistical Analysis Software (version 29.0.1.0) and compared using a *p*-value *α* < 0.05.

### Quality improvement interventions

The NICU bCPAP workgroup met monthly during Period 3 through a virtual platform to discuss unit staff feedback, track patient outcomes and review SPC charts. Change ideas were generated and selected for PDSA cycles to address identified problems and roadblocks and to improve patient outcomes.

### Ethical considerations

Ethical clearance to conduct this study was obtained from the Amoud University Institutional Review Board on 21 August 2023. All procedures performed in studies involving human participants were in accordance with the ethical standards of the institutional and/or national research committee and with the 1964 Helsinki Declaration and its later amendments or comparable ethical standards. Verbal informed consent for this QI initiative was obtained from the parents or legal guardians of participants involved in the study. The reason for the absence of written consent is due to resource limitations and hospital-specific policies and practices.

## Results

During the baseline period (Period 1), 10 infants were evaluated on the previously employed bCPAP device. Demographic data, timing of bCPAP initiation, indication for initiation of CPAP, and selected process, outcome, and balancing measures are displayed in Table 1 for the baseline (Period 1) and study intervention cohort (Period 3).

The first staff training took place in Period 2, during which 100% (7/7) of NICU nurses, 100% (11/11) of FM residents (4 post-graduate years [PGY-1], 2 PGY-2, 5 PGY-3) and 100% (3/3) medical interns completed the on-site training. The medical interns rotated monthly, and thus, we did not capture the complete cohort of medical interns. The staff were trained on respiratory education topics including non-invasive ventilation, neonatal respiratory distress, bCPAP setup, introduction to QI methodology, introduction to infection control, and troubleshooting of common problems with non-invasive ventilation. Following the completion of the six topics, a multiple-choice post-training assessment was administered to the 11 FM residents. The pass rates varied across the six topics covered, with three topics achieving a pass rate of 100%, two topics attaining a pass rate of 92%, and one topic achieving a pass rate of 80%. Score reports are summarised in Table 1.

During the study period (Period 3), 45/45 (100%) of infants were started on bCPAP during the first 3 days of life, with 42/45 (93%) initiated in the first day of life (DOL), 1/45 (2%) initiated in the second DOL and 2/45 (4%) initiated in the third DOL (Table 1). None of the patients were initiated on bCPAP in the delivery room because no bCPAP setup was available inside the delivery room. The primary indications to initiate bCPAP were RDS, TTN, MAS, pneumonia, sepsis, apnoea, hypoxic-ischaemic encephalopathy (HIE) and cardiac disease (Table 1).

**FIGURE 3:**
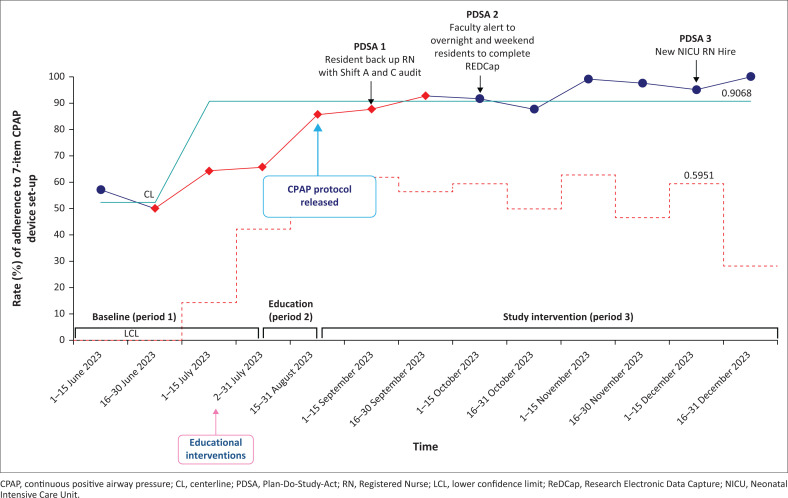
Statistical process control u-chart demonstrating the process measure for rate (%) of adherence to the 7-item continuous positive airway pressure device setup.

**FIGURE 4:**
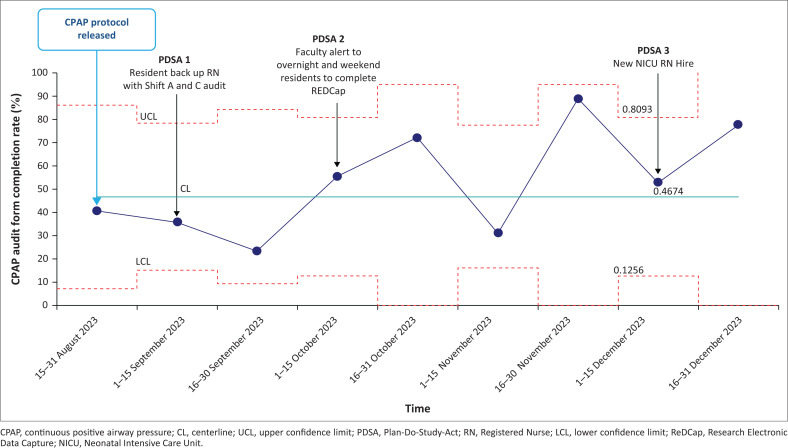
Statistical process control *u*-chart demonstrating the process measure for rate (%) of continuous positive airway pressure audit form completion by the bedside nursing staff.

During the study period (Period 3), 45 infants were initiated on the locally designed bCPAP device. The bCPAP safety checklist audit form was completed at a rate of 47% (122/261) for the three nursing shifts daily while the patients remained on bCPAP. The 2-week bCPAP safety audit completion rates are demonstrated in Figure 4. We achieved our study aim to improve respiratory distress by providing effective bCPAP by demonstrating an increase in the adherence rate to the 7-item bCPAP device setup from a baseline of 52% to 91% (Figure 3).

**FIGURE 5:**
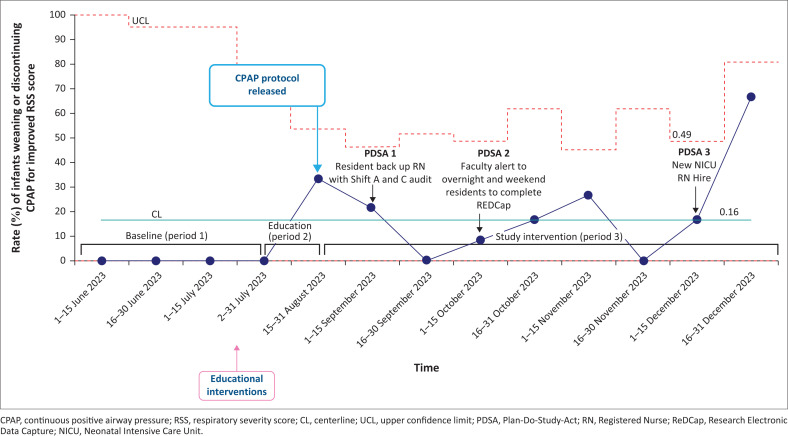
Statistical process control p-chart demonstrating the outcome measure for rate (%) of infants weaning or discontinuing continuous positive airway pressure for improved respiratory severity score.

During the baseline data collection period, 100% (10/10) of infants remained on bCPAP due to persistent signs and symptoms of respiratory distress (Figure 5). Following the study protocol implementation, the rate of infants weaning or discontinuing bCPAP for improved RSS increased from 0% to 18% but did not meet process change criteria (Figure 5). Of those infants who were weaning or discontinuing bCPAP support, we compared the indications for initiation of bCPAP. We observed that infants initiated on bCPAP for TTN were more likely to be weaning on bCPAP support, whereas infants initiated on bCPAP for RDS were less likely to be weaning on bCPAP support (Table 1). There was no statistically significant difference noted for the remaining indications for initiation of bCPAP (Table 1).

The total mortality in the unit during the baseline period was 11/35 (31%). Seven out of 10 (70%) infants receiving bCPAP treatment died during the baseline period. The indications for the bCPAP in the deceased patients were RDS 3/7 (42%), MAS 2/7 (29%) and HIE 2/7 (29%). The total mortality in the unit during the implementation period was 30/124 (24%). Of the infants included in the study, 6/45 (13%) infants died during the study period. Of those deceased, 4/6 (67%) were premature with Gestational Age (GA) of < 37 (29, 30, 31 and 35 weeks) and had RDS listed as the primary indication for initiation of CPAP. Of the two term infants who died, one patient’s indication for bCPAP initiation was listed as HIE, and the other infant had sepsis as the primary indication for initiation of bCPAP.

**FIGURE 6:**
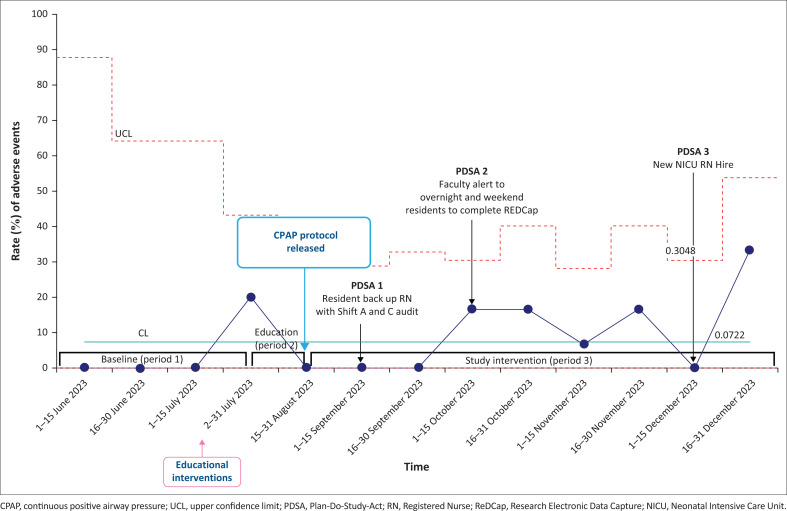
Statistical process control u-chart demonstrating the balancing measure for rate (%) of adverse events (nasal irritation, columella breakdown, pneumothorax).

Throughout the implementation time, six bCPAP machines were maintained and none had any documented mechanical issues that would have restricted availability. There was no increase in adverse event rates (rate of air leak, nasal columella breakdown and nasal irritation) (Figure 6).

### PDSA 1

Gap: After 4 weeks from the start of implementation, residents noticed that bedside nurses did not have time to complete the audits because of the high census rate.

Change idea: Interns and residents helped to complete the safety audit checklist during morning and evening rounds.

Result: After this step, the bCPAP safety audit completion rate improved to 77% in the remaining weeks (Figure 4).

### PDSA 2

Gap: Low staffing coverage (two FM residents covering the entire hospital) on overnight and weekend shifts led to low completion rates of the REDCap survey. The residents were having difficulty completing the data entry form on nights and weekends.

Change idea: To close this gap, faculty and resident research leads made announcements at morning check-out daily to ensure overnight residents had completed the REDCap form for overnight admissions. During weekends, on-call residents from the previous night completed the data entry form before they left the hospital in the morning.

Results: After the initiation of announcements and reminders from the faculty physicians, we tracked REDCap survey completion overnight and on weekend days and did not see any improvement in rates of completion during these selected times.

### PDSA 3

Gap: Historically, there has been continuous staff turnover in the hospital. Any new NICU staff hires during the study period were not part of the initial education training implemented during Period 2. Fortunately, there was minimal staff turnover during the intervention period (Period 3) and only one new shift C nurse was hired in December 2023.

Change idea: The train-the-trainer model was deployed where previously trained nurses and resident physicians conducted a bCPAP education training session for the new hire.

Result: The new NICU nurse completed the new hire bCPAP training conducted by previously trained nurses and the on-call physicians. Reassuringly, no decline in the rate of adherence to the bCPAP setup (Figure 3) or rate of safety audit form completion (Figure 4) was noted after the new nurse hire was trained.

## Discussion

Respiratory distress at birth is a leading contributor to neonatal morbidity and mortality worldwide.^9,34,35,36,37^ Many LMICs lack access to advanced ventilation support measures that are available in higher-resourced settings, such as airway devices (e.g. mechanical ventilator, endotracheal tube, supraglottic airway device), supplemental oxygen, medications (e.g. caffeine, surfactant), procedural skills (e.g. intubation procedural proficiency) and systems-based infrastructure (e.g. neonatal monitoring unit, adequate staffing).^38,39^ Low-cost, non-invasive bCPAP therapy is often the mainstay of treatment for neonates with respiratory distress in lower-resourced settings, including neonates admitted to the BRH NICU.^40^ A rudimentary bCPAP device was operational in the NICU before the start of the study. However, baseline data revealed several barriers to effective CPAP delivery, and no standardised clinical protocol existed to evaluate respiratory distress severity or guide bCPAP initiation, escalation, de-escalation and discontinuation. In the 6 months of data tracking, this single-site QI initiative demonstrated increased compliance with the 7-item bCPAP setup checklist, increased rates of bCPAP safety audit completion, and increased rates of infants able to wean or discontinue CPAP therapy.

While no significant adverse events were observed in this study, a study that implemented bCPAP in a Malawian paediatric unit identified nasal prong-associated complications and nasal septal injury, emphasising the importance of appropriate interfaces, nurse-to-patient ratios and continuous training.^41^ Similarly, a cluster-randomised trial in Ethiopia demonstrated the benefits of bCPAP in reducing treatment failure and mortality compared to low-flow oxygen, with no significant adverse effects in settings with adequate staffing and supervision by paediatricians.^42^ Both studies emphasise that structured protocols, such as checklists for device setup, and continuous monitoring are crucial for minimising risks. However, barriers like equipment availability, staff shortages and infrastructure limitations remain common challenges across settings, reinforcing the need for cost-effective, context-adapted solutions.

The development of a locally sustainable bCPAP programme requires appropriate equipment, staff knowledge and system-based support. System-based needs include but are not limited to hospital-based financing, facility maintenance (e.g. supply of electricity, disinfection materials) and hospital administration support.^43^ Prior to the implementation of this QI initiative, local physician leaders had already addressed some hospital-based infrastructure barriers to supporting neonates in respiratory distress. They secured funding and staffing to establish the region’s first NICU in 2017 and worked closely with hospital administration to allocate a dedicated panel of bedside nurses to staff the unit. Building upon these previous successes, staff knowledge and skills were prioritised as a key component of this QI initiative to ensure effective device utilisation and develop a mechanism for ongoing staff education.

### Strengths

This study demonstrated several notable strengths. First was the incorporation of the extensive 2-week education period for staff skills training. This approach was instrumental in establishing a standardised approach to evaluating infants experiencing respiratory distress and determining the necessity of bCPAP treatment by utilising a validated scoring system, the RSS. Additionally, it provided guidance on how to effectively disinfect and store equipment once infants were no longer on bCPAP. The educational training materials were made available to all faculty and nursing leadership after the training period to support ongoing educational support for using a ‘train-the-trainer’ model.^44^ The group of nurses and providers who completed the initial training now provide on-site training for new nursing hires, interns and residents to promote programme sustainability. Following the study’s completion, data was shared with NICU staff and dissemination to hospital leadership to advocate for retaining the nurses trained in the initial educational interventions.

Another strength of this study was the incorporation of a pressure testing device for improved safety. Given the variability in oxygen concentrator flow, this apparatus enabled bCPAP pressure testing at the patient interface. The 2-week education period also included a critical evaluation of available and scarce resources needed for the bCPAP device and clinical protocol. Modifications and adaptations to published protocols were made based on real-time feedback from local staff to increase the feasibility and sustainability of the QI team’s change ideas. The use of locally available products was prioritised over internationally supplied equipment. The disinfection system components (i.e. buckets, brushes and gloves) were all obtained from the local market, and the chlorine disinfectant solution was supplied by the hospital. Furthermore, the disinfection system was purposefully designed to enable re-utilisation of individual components of the bCPAP system, mitigating the impact of limited bCPAP equipment availability, which will be explained more in the limitations. Lastly, the scalability of the bCPAP model, demonstrated by the results, suggests that this approach could be successfully implemented in other low-resource settings, provided similar protocols and training programmes are put in place.

### Limitations

Despite these significant improvements, systems-based barriers to optimisation of the BRH bCPAP programme remain. The NICU is located in a separate building from the labour and delivery unit, thus requiring outdoor exposure to transfer to the NICU with no ability to provide bCPAP therapy during transport. While the development of a transportable bCPAP system and inter-facility transport device was out of the scope of the current QI initiative, it will be an essential gap to address as the project’s next steps. Owing to geographic barriers, sourcing a sustainable supply of select bCPAP equipment (e.g. RAM cannula and large bore tubing) not available through local medical device suppliers will need to be sustained through international shipping to available ports.

Data tracking is a vital component of characterising gaps and tracking QI goals to ensure that changes in clinical management lead to improvement in process and outcome measures.^45,46^ This multidisciplinary QI team determined that the bCPAP safety audit was best collected by paper audit, for ease of use by bedside nursing staff. The disadvantage is the potential for data loss if the paper audit is not returned to the patient’s bedside chart. In contrast, the comprehensive demographic and study measure audit form was developed as a secure electronic data entry form. This afforded real-time accessibility of recorded information to the US-based team; however, given there were no computers in the NICU, data collection required the use of personal smartphones to access the online survey. This potentially limited data collection if the provider did not have access to a mobile device or internet connection on daily rounds. Along with this, variability in survey completion during times of high-volume bCPAP use was observed. A new PDSA cycle was initiated in response to this feedback where either the medical intern or senior FM resident would complete the bCPAP safety checklist during morning and evening NICU rounds in periods of high census to ensure bedside nursing staff did not neglect clinical care duties due to data entry. While this adjustment improved the situation, it underscores the challenges of maintaining consistent data collection during busy periods in the NICU, which can be a target for future studies.

To standardise the assessment of respiratory distress for this study, the Silverman Anderson RSS was chosen over the Downes score.^12^ When assessing the capabilities of this study site, the Silverman Anderson RSS allowed for easier use by healthcare workers with differing levels of English proficiency given the incorporation of visual respiratory distress signs rather than written descriptions. Variation in assessment skills continued, as we noted some infants were initiated on therapy with lower RSS. Only upon review of the patient records after completion of the study was it discovered that some RSS values were falsely low due to inaccurate reporting by staff. This should have prompted a refresher training to improve the misclassification; however, it was not appropriately identified by the QI team during the study period. Additionally, it is important to highlight that apnoea is not accounted for in the RSS. Therefore, those infants with a low RSS and apnoea were appropriately initiated on CPAP therapy.

Ongoing staff shortages and high turnover rates at BRH emerged as a persistent challenge, thereby impeding project continuity and standardisation of bCPAP training and data collection procedures among NICU personnel. We attempted to address these gaps in PDSA cycles 2 and 3. However, in PDSA cycle 2, we did not observe an improvement in rates of REDCap data audit completion overnight or on weekends after the initiation of reminders from the faculty physicians. This was not surprising given no change in the staffing model was implemented. This was outside the scope of feasibility for this QI initiative, given staff duty hour limitations and financial barriers to hiring new staff. This will be a potential target for future initiatives.

The X-ray machine at BRH is not mobile and thus unavailable for use in the NICU. Furthermore, the cost of obtaining an X-ray is unaffordable for many NICU patients. Lack of access to the X-ray machine may have led to a decreased diagnosis of air leak or pneumothorax. Variability in oxygen concentrators and manual modifications to water bottles to fit the expiratory tubes could have introduced inconsistencies in bCPAP delivery. In addition, the equipment used for this locally designed setup was off-label, which limits our ability to draw conclusions regarding the device’s safety. Future studies should exercise caution when replicating similar setups without stringent safety testing protocols in place.

Our QI team acknowledges that while the local adaptation of certain protocol elements likely promoted the site-specific study success, the customisation of this bCPAP system to the specific needs of a single centre limits the generalisability of this study. The small sample size of this study is also a significant limitation. As a result of the sample size limitations, there is not enough data to make a direct link of CPAP to a decrease in neonatal mortality. However, there are potential links established in larger studies.^15,20^ Future efforts to address these study limitations include a focus on logistical and staffing challenges, continued process and outcome measure monitoring, and programme expansion to nearby hospitals to evaluate the scalability of the findings.

### Implications and recommendations

This study outlines commonly encountered barriers to adherence with the standard of care (bCPAP) for neonatal respiratory distress and provides a framework for closing gaps in best practices in low-income countries. Strong recommendations based on our study observations include the need for focused staff training to address skill and knowledge gaps in device maintenance (setup, pressure delivery, patient interface challenges) and respiratory assessment. The use of a ‘train-the-trainer’ model promoted programme sustainability by allowing trained NICU staff to pass on their knowledge to new hires, ensuring continuity of clinical practice with minimal financial burden. We also highlight the importance of locally sourced materials with a plan for equipment disinfection to overcome supply chain barriers and improper reprocessing that can lead to equipment gaps. We advise QI teams to explore hospital-specific resources and local equipment availability to customise their adapted devices to ensure site-specific appropriateness. We do not recommend the use of non-commercial bCPAP devices in centres that do not have the capability of pressure testing the device. Quality improvement teams may need to test several change ideas to achieve their study-specific aim and employ ongoing data audits to ensure maintenance of desired practice change is observed.

## Conclusion

This single-centre study describes one QI team’s approach to identifying and reducing neonatal respiratory distress through bCPAP therapy in a low-resource setting. While bCPAP is an inexpensive and highly effective form of non-invasive ventilation, its successful implementation depends on staff knowledge, equipment maintenance and close clinical supervision. Clinical protocol implementation to standardise bCPAP is well suited to time series analysis to determine whether clinical practice changes are effective in closing local gaps in adherence to the standard of care for neonatal respiratory distress.
